# Modelling foetal exposure to maternal smoking using hepatoblasts from pluripotent stem cells

**DOI:** 10.1007/s00204-017-1983-0

**Published:** 2017-05-16

**Authors:** Baltasar Lucendo-Villarin, Panagiotis Filis, Madeleine J. Swortwood, Marilyn A. Huestis, Jose Meseguer-Ripolles, Kate Cameron, John P. Iredale, Peter J. O’Shaughnessy, Paul A. Fowler, David C. Hay

**Affiliations:** 1 Medical Research Council Centre for Regenerative Medicine, University of Edinburgh, 5 Little France Drive, Edinburgh, EH16 4UU Scotland, UK; 2 Institute of Medical Sciences, University of Aberdeen, Foresterhill, Aberdeen, AB25 2ZD UK; 3 Department of Forensic Science, College of Criminal Justice, Sam Houston State University, Huntsville, TX USA; 4 University of Maryland School of Medicine, Baltimore, MD 21201 USA; 5 University of Bristol, Senate House, Tyndall Avenue, Bristol, BS8 1TH UK; 6 Institute of Biodiversity, Animal Health and Comparative Medicine, University of Glasgow, Glasgow, G61 1QH UK

**Keywords:** Maternal smoking, Human development, Pluripotent stem cells, Hepatocytes, Apoptosis, Necrosis

## Abstract

The liver is a dynamic organ which is both multifunctional and highly regenerative. A major role of the liver is to process both endo and xenobiotics. Cigarettes are an example of a legal and widely used drug which can cause major health problems for adults and constitute a particular risk to the foetus, if the mother smokes during pregnancy. Cigarette smoke contains a complex mixture of thousands of different xenobiotics, including nicotine and polycyclic aromatic hydrocarbons. These affect foetal development in a sex-specific manner, inducing sex-dependant molecular responses in different organs. To date, the effect of maternal smoking on the foetal liver has been studied in vitro using cell lines, primary tissue and animal models. While these models have proven to be useful, poor cell phenotype, tissue scarcity, batch-to-batch variation and species differences have led to difficulties in data extrapolation toward human development. Therefore, in this study we have employed hepatoblasts, derived from pluripotent stem cells, to model the effects of xenobiotics from cigarette smoke on human hepatocyte development. Highly pure hepatocyte populations (>90%) were produced in vitro and exposed to factors present in cigarette smoke. Analysis of ATP levels revealed that, independent of the sex, the majority of smoking derivatives tested individually did not deplete ATP levels below 50%. However, following exposure to a cocktail of smoking derivatives, ATP production fell below 50% in a sex-dependent manner. This was paralleled by a loss metabolic activity and secretory ability in both female and male hepatocytes. Interestingly, cell depletion was less pronounced in female hepatocytes, whereas caspase activation was ~twofold greater, indicating sex differences in cell death upon exposure to the smoking derivatives tested.

**Electronic supplementary material:**

The online version of this article (doi:10.1007/s00204-017-1983-0) contains supplementary material, which is available to authorized users.

## Introduction

The liver is the body’s second largest organ playing a major role in the processing of xenotoxicants, which include alcohol, drugs and environmental pollutants. Cigarettes are an example of a widely used drug which can cause major health problems for adults, and constitute a particular risk to the developing foetus. Cigarettes contain a complex mixture of over 7000 different compounds (Rodgman et al. 2009) which include nicotine and the polycyclic aromatic hydrocarbons (PAHs). Nicotine is primarily metabolised by cytochrome P450 2A6 (CYP2A6) in the liver (Benowitz et al. 1994) into several metabolites, of which cotinine represents approximately 70–80% (Messina et al. 1997). PAHs are incomplete combustion products first identified as carcinogenic constituents of coal tar (Phillips 1983) and charcoal-grilled foods (Phillips 1999; Boström et al. 2002; Rodgman et al. 2009). PAHs are also detected in placental tissues and umbilical cord blood of smokers (Perera et al. 2005a; Al-Saleh et al. 2013) reaching the foetal liver from the maternal circulation. This exposes the developing foetus to harmful agents and leads to corresponding changes in gene expression (O’Shaughnessy et al. 2011).

In addition to toxicant exposure, smoking also disrupts foetal oxygen and carbon monoxide balance which can cause harmful effects, including impaired growth, premature birth, hormonal imbalances, increased predisposition to metabolic syndrome, liver disease and even death (Chen et al. 2006; Harvey et al. 2007; Rogers 2009; Mamsen et al. 2010; Fowler et al. 2011, 2014; Hackshaw et al. 2011; Högberg et al. 2012; Behl et al. 2013; Filis et al. 2015). Moreover, it has been reported that maternal smoking affects the foetus in a sex-specific manner. For example, male offspring possess a higher risk of developing conduct disorders, whereas female offspring are predisposed to developing weight disorders and drug dependence (Weissman et al. 1999; Chen et al. 2006). In addition, maternal smoking induces sex-dependant molecular responses in the reproductive organs and the liver of the developing foetus (Fowler et al. 2008; O’Shaughnessy et al. 2011; Drake et al. 2015).

To date, the effect of maternal smoking on the foetal liver has been studied in vitro using cell lines, primary tissue and animal models (Neumann 1986; Rao et al. 1988; Cho et al. 2006, Choi et al. 2015; Baxter 2009; Sanchez et al. 2011; Van Kesteren et al. 2013; Williams et al. 2014). While these models have proven to be informative, the scarcity of human tissue, the rapid loss of cell phenotype, batch-to-batch variation and species differences have led to difficulties in data extrapolation toward the human. Moreover, the mature nature of primary cells used in vitro impairs the study of foetal development ‘in the dish’. In contrast to the above sources, human hepatocytes derived from pluripotent stem cells have been proven to represent a reliable human model to study liver biology in detail (Szkolnicka et al. 2014, 2016; Villarin et al. 2015). To study the disruptive effects of smoking on human development, we have employed this renewable cell model. Pluripotent stem cell derived hepatoblasts were produced at scale from male and female cell lines. Following this, hepatocyte differentiation was performed in the presence of cotinine and PAHs and this led to sex-specific changes in cell biology.

## Methods and materials

### Cell culture and differentiation

H9 and Man12 human embryonic stem cells (hESCs**)** identity was confirmed using short tandem repeat typing. hESCs were cultured and differentiated as previously described (Cameron et al. 2015). Maintenance of hESCs was performed on pre-coated laminin 521 (Biolaminin) in mTeSR1 (STEMCELL Technologies) in a humidified 37 °C, 5% CO_2_ incubator. For differentiation, hESCs were plated onto a pre-coated blend of laminins 521 and 111 (at a 1:3 ratio). Differentiation was initiated at 40% confluence by replacing serum-free medium with endoderm differentiation medium: RPMI 1640 containing 1× B27 (Life Technologies), 100 ng/mL Activin A (PeproTech), and 50 ng/mL Wnt3a (R&D Systems). The medium was changed every 24 h for 72 h. On day 3, endoderm differentiation medium was replaced with hepatoblast differentiation medium, and this was renewed every second day for a further 5 days. The medium consisted of knockout (KO)-DMEM (Life Technologies), Serum replacement (Life Technologies), 0.5% Glutamax (Life Technologies), 1% non-essential amino acids (Life Technologies), 0.2% β-mercaptoethanol (Life Technologies), and 1% DMSO (Sigma). On day 8, differentiating cells were cultured in the hepatocyte maturation medium HepatoZYME (Life Technologies) containing 1% Glutamax (Life Technologies), supplemented with 10 ng/mL hepatocyte growth factor (PeproTech) and 20 ng/mL oncostatin M (PeproTech). On day 10 maturation medium was replaced with HepatoZYME supplemented with and without smoking derivatives (Sigma-Aldrich) for a further 8 days, with media replaced every 48 h.

### Immunofluorescence

Cell cultures were fixed in 100% ice-cold methanol at −20 °C for 30 min. Subsequently, fixed cells were washed twice with PBS at room temperature. Cell monolayers were blocked with 0.1% PBS-Tween containing 10% BSA for 1 h, and the monolayers were incubated with primary antibodies diluted in PBS-0.1% Tween/1% BSA at 4 °C overnight (Supplementary Table 1). The following day, the primary antibody was removed, and the fixed monolayers were washed three times with PBS-0.1% Tween/1% BSA. Following this, the cells were incubated with the appropriate secondary antibody diluted in PBS/0.1% Tween/1% BSA for 1 h at room temperature and washed three times with PBS. Cultures were then mounted with PermaFluor aqueous mounting medium (Thermo Scientific) and counterstained with NucBlue Hoechst 33342 (Sigma-Aldrich). The cells were imaged with an Axio Observer Z1 microscope with LD PlanNeoFluar objective lenses (Carl Zeiss). This microscope was coupled to a Zeiss AxioCamMR3 camera used for image acquisition. The images were captured using a Zeiss Axiovision SE 64 Rel 4.8 and analysed using Zeiss Axiovision software version 4.9.1.0. The percentage of positive cells (±standard deviation) was estimated from at least eight random fields of view.

### Albumin and α-fetoprotein ELISA

hESC-derived hepatocyte protein secretion was measured by ELISA. Alpha-fetoprotein and albumin production was quantified using commercially available kits from Alpha Diagnostic International. The different media were collected at the day 18 in the differentiation process. Samples were run in duplicate and measured on a FLUOStar Omega multi-mode microplate reader (BMG Labtech). Protein production was expressed as either nanogram or microgram of protein per milliliter of medium per 24 h and per milligram of cellular protein [determined by the bicinchoninic acid (BCA) assay, Pierce] or as percentage of secretory capacity normalised to the vehicle control. Levels of significance were measured by Student’s *t* test. The experiments are representative of five biological replicates.

### Cytochrome P450 assays

CYP3A and CYP1A2 activity was measured in hepatocytes at Day 18 using pGlo technology (Promega) in accordance with the manufacturer’s instructions. CYP activity was expressed as either relative light units (RLUs) per milliliter of medium per milligram of protein (determined by the BCA assay, Pierce), or as a percentage of CYP activity normalised to the vehicle control. Levels of significance were measured by Student’s *t* test. The experiments are representative of five biological replicates.

### Cell health assays

Cell health was assessed measuring ATP production and Caspase 3/7 activity at Day 18 employing pGlo technology (Promega) in accordance with the manufacturer’s instructions. Levels of expression of both markers were expressed as percentage of relative light units (RLUs) per milliliter of medium and normalised to the vehicle control. Levels of significance were measured by Student’s *t* test. The experiments are representative of five biological replicates.

### Detection of smoking derivatives in foetuses from smokers and non-smokers

Cotinine was measured using LC–MS/MS methodology as follows. Cotinine and the internal standard ^2^H_3_-cotinine were dissolved in methanol and diluted in pooled human plasma to give calibration standards in the range 1.5–500 ng/mL. Quality control samples were prepared in pooled human plasma at 2.5, 250 and 450 ng/mL cotinine. To a 10 µL aliquot of plasma, 10 µL (2 ng) of IS was added and 250 µL 0.1% formic acid in water. After mixing, the samples were kept on ice for 15 min. Following centrifugation at 14,800 rpm for 15 s, the plasma samples were applied to BondElut Plexa PCX cartridges (30 mg/1 mL, Crawford Scientific, UK) that had been pre-conditioned and equilibrated using 0.5 mL of methanol and 0.5 mL of 0.1% formic acid in water. The cartridges were washed with 0.5 mL 0.1% formic acid in water followed by 2 × 0.5 mL 95/5 methanol/0.1% formic acid in water and cotinine and the IS eluted with 0.5 mL 95/5 methanol/ammonium hydroxide. The eluate was evaporated to dryness under nitrogen at room temperature and the residue re-suspended in 60 µL 50/50/0.1 water/methanol/formic acid. Following centrifugation at 14,800 rpm for 5 min, 5 µL of the supernatant was injected onto the chromatograph. Chromatography was performed on a Thermo Surveyor (Thermo Scientific, UK) system using a 150 × 2.1 mm ACE 3µ C18-AR column (Hichrom, UK) maintained at 50 °C. The mobile phase consisted of 0.1% ammonium acetate (A) and methanol (B) and elution achieved with a linear gradient over 3 min from 10 to 100% B with a hold of 1 min at 100% B. The flow rate was 200 µL/min and the samples were maintained at 4 °C in the autosampler. Total run time was 8 min. A Thermo TSQ Quantum triple quadrupole mass spectrometer was used in positive electrospray ionisation mode for the detection of cotinine. Quantification was performed using single reaction monitoring (SRM) scan mode using the following transitions: cotinine *m*/*z* 177.0–80.1 and ^2^H_3_-cotinine *m*/*z* 180.0–80.1. Flow injection analysis was used to optimise the MS/MS conditions as follows: spray voltage 4000 V, sheath gas pressure 60, auxiliary gas pressure 0, capillary temperature 375 °C, skimmer offset −10 V, collision pressure 1.7 mTorr and collision energy 25 V. Instrument control and peak integration and quantification were performed using Thermo Xcalibur software (v. 2.0.7 SP1). Weighted least squares linear regression with a weighting factor of 1/X was used to quantify the cotinine concentration in unknown samples by comparing peak area ratios (analyte/IS) with those obtained from a multi-level calibration standard curve. PAHs in human foetal livers were quantified in Fowler et al. (2014) and the results presented here in are in different format for display purposes. The collection of foetal material (Fowler et al. 2008) was approved by the National Health Service Grampian Research Ethics Committees (REC 04/S0802/21).

### Cell count

Following compound exposure, cells were washed with 100 μL/well of 1×HBSS (Invitrogen) and fixed with 50 μL of 4% (wt/vol) paraformaldehyde (PFA) for 20 min at room temperature. Cells were permeabilized with 50 μL/well of 0.1% (vol/vol) Triton X-100 (Sigma-Aldrich for 15 min), followed by a wash with 100 μL/well of 1×HBSS and an incubation with 50 μL/well of a solution containing 2 drops/mL of NucBlue Live ReadyProbes^®^ Reagent (Molecular Probes) in 1×HBSS for 5 min at room temperature. Following incubation, a final wash of 100 μL/well of 1×HBSS was performed. Fluorescent images were acquired using the Operetta high content analysis system with the Harmony High-Content Imaging and Analysis Software (PerkinElmer). 20 fields of view were acquired across the well to obtain an average representation of the well. Nuclei were quantified using the Acapella Image Analysis software (PerkinElmer, version: 4.1.1.118082™). The experiments are representative of five biological replicates.

### Statistical analysis

Unless indicated, all data were obtained from at least five biological replicates and are presented by Mean ± standard deviation (SD). The difference between control and treatment groups were tested by Student’s *t* test where *P* < 0.05 is denoted as *, *P* < 0.01 is denoted as ** and *P* < 0.001 is denoted as ***.

## Results

### Measuring foetal exposure to smoking-derived contaminants

**Fig. 1:**
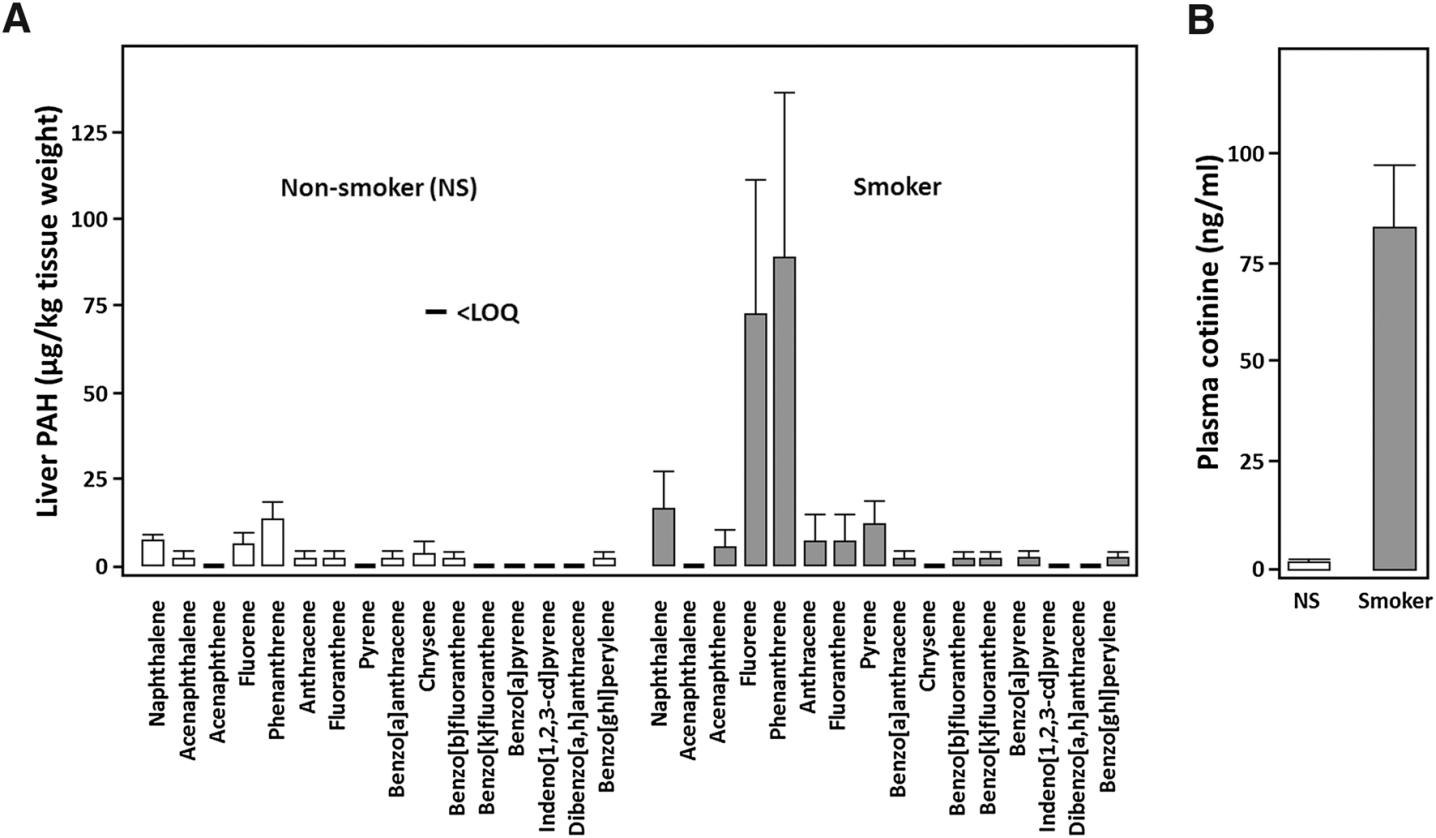
Concentrations of cigarette smoke derivates in the second trimester human foetus. **a** Polycyclic aromatic hydrocarbons (PAHs) in livers from 10 control and 12 smoke-exposed human foetuses. PAHs in human foetal livers were quantified in Fowler et al. (2014) and the results presented here are in different format for display purposes. **b** The predominant bioactive metabolite of nicotine, cotinine, in plasma from 16 control and 22 smoke-exposed foetuses

The active components of cigarette smoke are well known (Rodgman et al. 2009) and for the purposes of these experiments we focused on cotinine, the major bioactive metabolite of nicotine, and polycyclic aromatic carbons (PAHs). Both cotinine and PAHs are significantly increased in the foetus by maternal smoking (Fig. 1). Following previous in vivo experimentation, we therefore hypothesised that these compounds represent a threat to the normal development of the foetal liver and we wished to model this in vitro using a reliable developmental model. To test this, we generated hepatoblasts and hepatocytes from male and female human embryonic stem cells (hESCs) using established methodology (Cameron et al. 2015).

### Generation of hepatoblasts and hepatocytes from male and female human embryonic stem cells

**Fig. 2:**
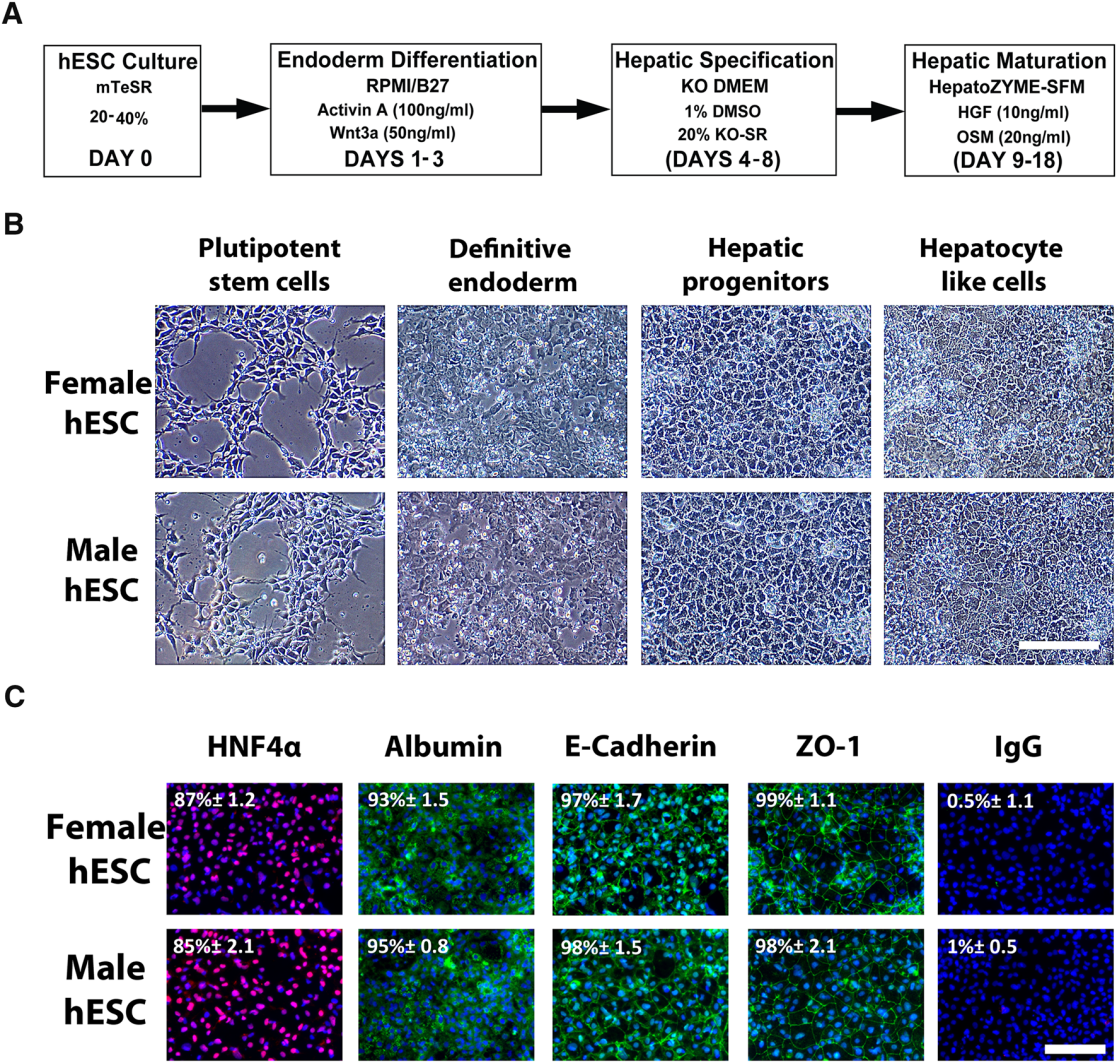
Characterisation stem cell derived hepatocytes. **a** Male and female human embryonic stem cells (hESC) were differentiated to hepatocytes employing a stepwise hepatocyte differentiation approach. **b** During differentiation, cells adopted different morphologies at each stage: definitive endoderm, hepatoblasts and hepatocytes. **c** Immunofluorescence was employed to examine the expression of the hepatocyte proteins HNF4α (*red*) and albumin (*green*), and epithelial markers E-cadherin (*green*) and Zona Occludens-1 (*green*). Morphological images were taken at ×10 magnification and scale bar represents 200 μm. For each condition eight random fields of view, containing at least 400 cells, were counted

Both hepatoblasts and hepatocytes were produced at scale from hESCs using a stagewise process (Cameron et al. 2015) to generate highly pure populations (Fig. 2a). During cellular differentiation, cells from both genders underwent similar morphological changes, culminating in typical hexagonal hepatocyte morphology (Fig. 2b). Further characterisation of the hepatocyte populations demonstrated that albumin was expressed in 93% of female and 95% of male hepatocytes. In these populations HNF4α was detected in 87 and 85% of female and male hepatocytes. To determine if hepatocytes were polarised we examined E-Cadherin and Zonal Occludin1 (ZO-1) expression. E-Cadherin was expressed in 97 and 98% of female and male cells, whereas ZO-1 expression detectable in 99 and 98% of female and male cells, respectively (Fig. 2c).

### Generation of functional hepatocytes from male and female human embryonic stem cells

**Fig. 3:**
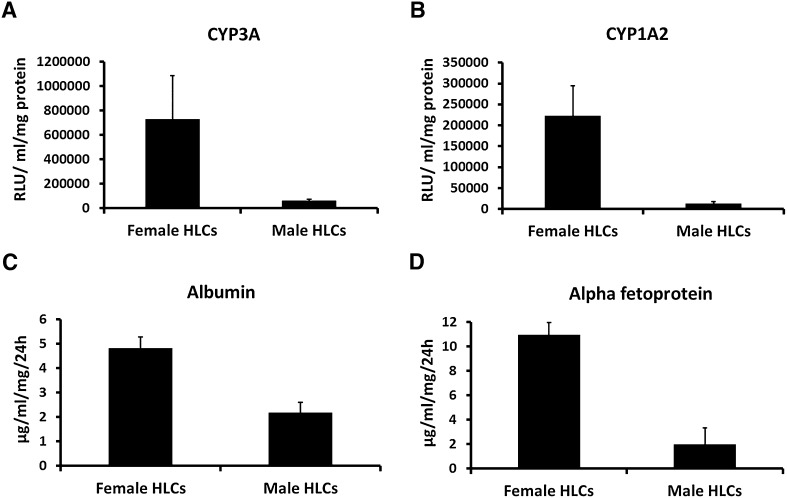
Stem cell derived hepatocytes display hepatocyte functions. Male and female hESCs derived hepatocytes were functionally characterised employing. **a**, **b** pGLO™ technology to study cytochrome p450 CYP3A and CYP1A2 function. **c**, **d** ELISA to measure the secretion of hepatocyte proteins albumin and alpha-fetoprotein. Levels of significance were measured by Student’s *t* test. The experiments are representative of five biological replicates

Following basic characterisation of morphology and gene expression, hepatocyte metabolic and secretory capacity was studied using pGlo™ and ELISA technologies. Hepatocytes from both sexes demonstrated appreciable levels of CYP1A2 and CYP3A activity (Fig. 3a, b). Cytochrome P450 activity was greater in female hepatocytes and aligned with a previous study (Villarin et al. 2015). Following this, albumin (ALB) and alpha-fetoprotein (AFP) secretion were measured. Female hepatocytes secreted 4.8 μg/mL/mg protein of ALB and 10.9 μg/mL/mg protein of AFP (Fig. 3c, d), whereas male hepatocytes secreted 2.1 μg/mL/mg protein of ALB and 2 μg/mL/mg protein of AFP (Fig. 3c, d). These experiments demonstrated that male and female hepatocytes displayed dependable performance and were suitable for the subsequent modelling experiments.

### Determining the sex differences in hepatocyte biology following exposure to cotinine and PAHs

Hepatocyte specification and maturation was performed in male and female hESCs lines in the presence or absence to smoking components. Cotinine at a concentration range of 1 to 300 nM, chrysene at a concentration range of 10 nM to 50 μM, fluorene at a concentration range of 10 nM to 100 μM, naphthalene at a concentration range of 10nm to 1 mM and phenanthrene, at a concentration range of 10 nM to 500 μM were used. Following 8 days exposure, cell health was determined by ATP production and caspase activity. Following this cell function was determined measuring CYP P450 3A and 1A2 activity. In order to measure the effectiveness of each component of cigarette smoke to inhibiting activity we used the half maximal inhibitory concentration (IC_50_).

Analysis of ATP levels revealed that, independent of the sex, the majority of smoking derivatives tested did not deplete ATP levels below 50%. The exception was phenanthrene, which reduced male ATP levels by 50% at 78 μM. Cell health was also studied by measuring caspase 3/7 activation in hepatocytes (Table 1; Supplementary Fig. 1 and 2). In these experiments we observed a sex-dependent response in caspase activation. While male and female hepatocytes were equally sensitive to continine, male hepatocytes were more sensitive to chrysene, fluorene and phenanthrene, whereas female hepatocytes were more sensitive to naphthalene. Subsequently, we studied the IC_50_ for CYP P450 following exposure to smoking derivatives (Table 1; Supplementary Fig. 1 and 2). Loss of CYP1A2 function was more pronounced in female hepatocytes following exposure to cotinine, chrysene and phenanthrene whereas male hepatocytes were more sensitive to fluorene. Of note, naphthalene did not reduce CYP P450 activity but, instead, induced CYP1A2 activity in male and female hepatocytes (Table 1). Analysis of the CYP3A function in response to the smoking derivatives also showed sex differences. Both male and female hepatocytes responded in a similar fashion to cotinine, whereas male hepatocyte function was more sensitive to chrysene, fluorene and naphthalene than female hepatocytes. Both male and female hepatocytes responded in a similar fashion to cotinine, whereas loss of male CYP3A function was less sensitive to chrysene and more sensitive to fluorene, naphthalene and phenanthrene than female hepatocytes.

**Table 1:** IC50 values of the tested compounds on cell health and cell metabolism

	IC_50_ ATP		Caspase 3/7 peak activity		IC_50_ CYP1A2		IC_50_ CYP3A	
	Female	Male	Female	Male	Female	Male	Female	Male
Cotinine	>300 nM	>300 nM	100 nM	100 nM	50 nM	100 nM	50 nM	50 nM
Chrysene	>50 μM	>50 μM	25 μM	5 μM	4.75 μM	55 μM	5 μM	10 μM
Fluorene	>100 μM	>100 μM	>100 μM	100 μM	50 μM	30 μM	34 μM	25 μM
Naphthalene	>1000 μM	>1000 μM	50 μM	1000 μM	Induced activity	Induced activity	700 μM	68 μM
Phenanthrene	>500 μM	78 μM	>500 μM	10 μM	10 μM	>500 μM	300 μM	58 μM

Male and female hepatocytes were exposed to the compounds for 8 days. Cell health was measured by analysing ATP production and Caspase 3/7 activity. Cell metabolism measured CYP1A2 and CYP3A activity

### Determining the sex differences in hepatocyte function following exposure to smoking derivatives

**Fig. 4:**
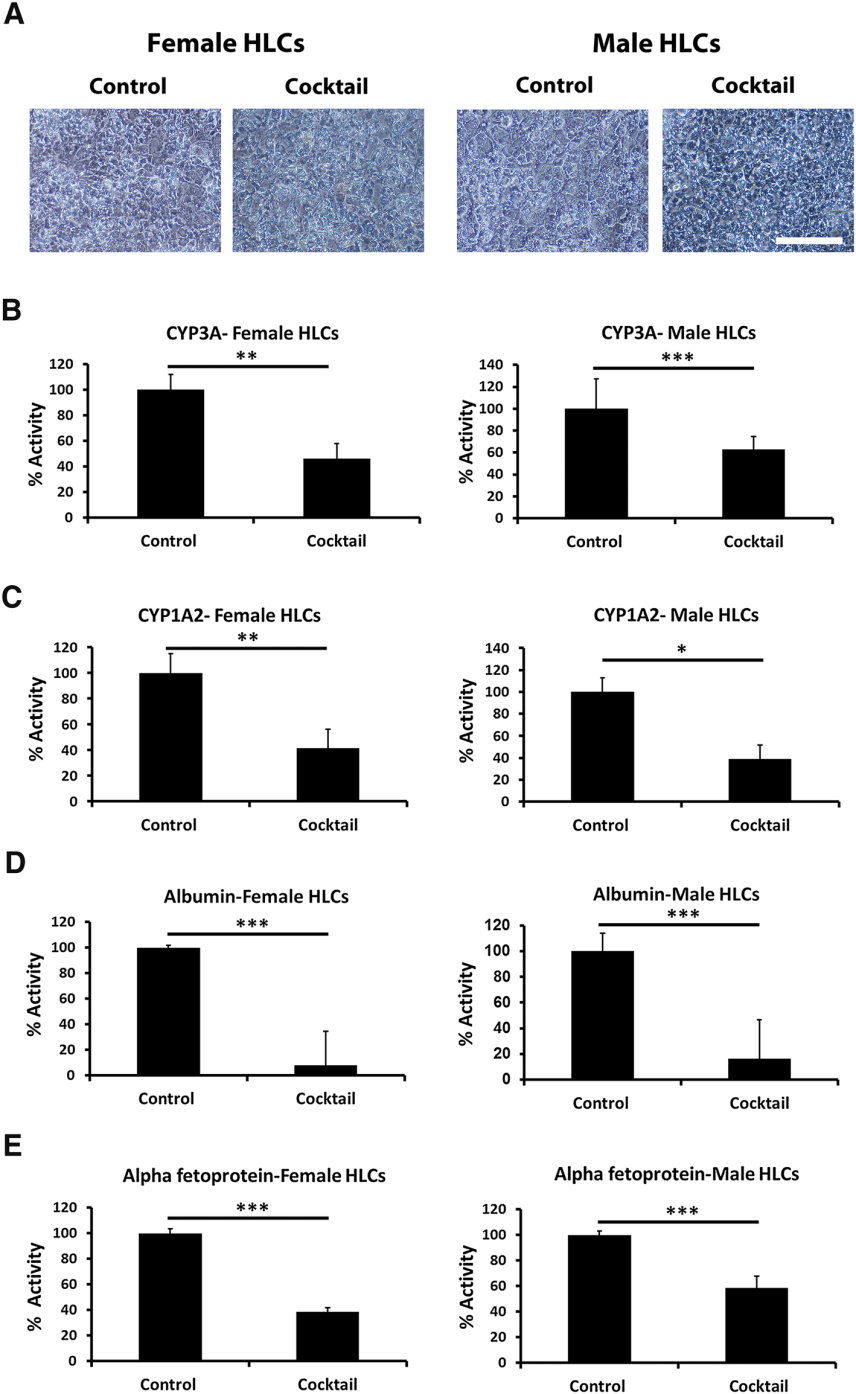
Cell morphology and metabolic activity following smoking component exposure. **a** Phase contrast images reveal a deterioration in the cell morphology in the presence of the mixture of drugs for 8 days compared with cells in the presence of the vehicle control. **b**, **c** pGLO™ technology was employed to measure cytochrome P450 activity. **d**, **e** ELISA was employed to study the secretion of albumin and alpha-fetoprotein. The images were taken at ×10 magnification and scale bar represents 200 μm. Levels of significance were measured by Student’s *t* test. The experiments are representative of five biological replicates

CYP3A is a major phase 1 enzyme family involved in the third trimester of human foetal development. To study the effect of a cocktail composed of cotinine and PAHs, we incubated hepatocytes with these additives at the IC_50_ values for CYP3A calculated in Table 1. Phase contrast images of the cells after incubation with the drug cocktail revealed morphological deterioration in both sexes (Fig. 4a). Descriptively, female hepatocytes lost hallmark hepatocyte features and displayed more fibroblast-like structures, whereas male hepatocytes exhibited a more rounded morphology. Cell function in both hepatocyte populations was also studied in detail. CYP3A activity was reduced by 54% in female hepatocytes and by 38% in male hepatocytes following exposure (Fig. 4b). This was in contrast to CYP1A2 activity which was reduced to similar extents in female and male hepatocytes (Fig. 4c). We also studied the effect that compound incubation had on protein secretion. In accordance with cell CYP P450 function, secretion of both ALB and AFP were reduced in male and female hepatocytes following exposure to the smoking derivatives (Fig. 4d, e).

### Determining the sex differences in cell biology following exposure to smoking derivatives

**Fig. 5:**
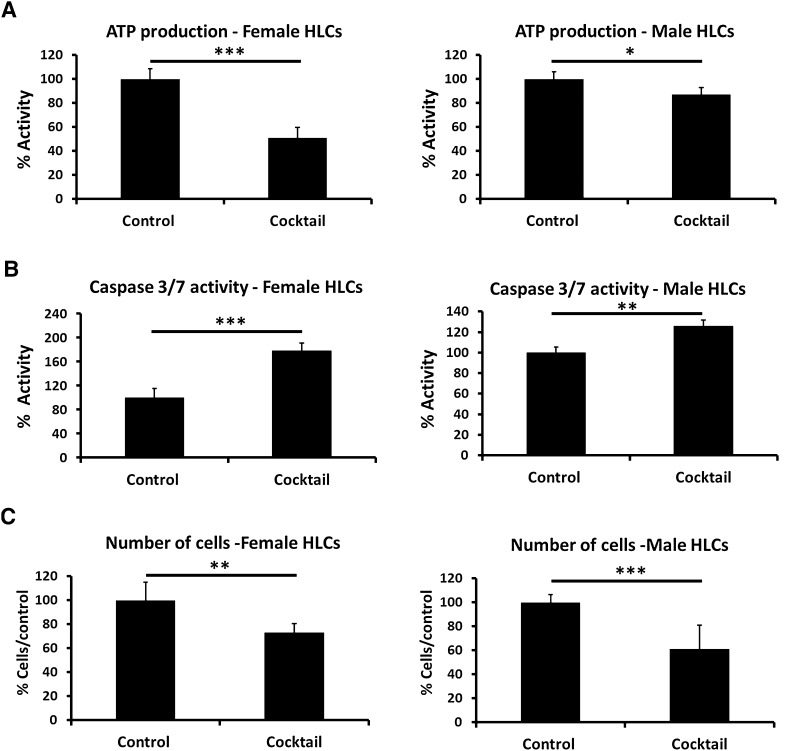
Measurement of the cell health in female and male hepatocytes following exposure to cocktail for 8 days. **a** Cell viability was studied by measuring the levels of ATP. **b** Cell apoptosis was measured using Caspase 3/7 activity. **c** Cell paint technology was employed to analyse the number of cells attached to the matrix

In addition to cell function, cell health was studied measuring ATP levels and caspase 3/7 activity. ATP levels were reduced to a greater extent in female hepatocytes than male hepatocytes (Fig. 5a). Whereas caspase activation was greater in female hepatocytes (~twofold increase) than male hepatocytes (~1.2 fold) (Fig. 5b). Following these experiments we examined the number of hepatocytes that remained in culture post exposure. Interestingly, male hepatocytes were depleted by 40%, whereas female hepatocytes were depleted by 30%. Taken together these data demonstrate that male hepatocytes were more likely detaching from the matrix and undergoing necrosis, whereas the female hepatocytes were undergoing cell dedifferentiation and apoptosis following expoure.

## Discussion

The ability to study the effects of maternal smoking on the unborn foetus has traditionally relied on material from elective terminations, animal models and a variety of cell lines. While these approaches have generated highly informative datasets, they do suffer from some significant drawbacks which include tissue scarcity, individual variability and loss of cell phenotype. Therefore, to study of the effects of the maternal smoking on human foetal liver development, renewable cell based models are required which can be delivered from defined genetic backgrounds. Encouragingly, pluripotent stem cell based liver models have proven to be effective in modelling human liver exposure to drugs in the past (Medine et al. 2013; Villarin et al. 2015; Szkolnicka et al. 2014, 2016). In these studies we have employed pluripotent stem cells to produce human hepatoblasts at scale and screen for developmental perturbation over an eight day time course.

Cigarettes contain a complex mixture of chemicals. These compounds pose a risk to foetal development which includes increased risk of intrauterine growth restriction, small-for-gestational age and preterm delivery amongst others (Perera et al. 1998, 2005a, b; Dejmek et al. 2000). To identify the major players for our modelling experiments we employed gas chromatography and mass spectrometry in foetal plasma and livers to identify specific cigarette smoke components. From these experiments we identified cotinine and four PAHs present in foetal circulation of mothers who smoked. We used this information to study the effects of those derivatives on hepatocyte differentiation from male and female hESCs. On the whole, exposure to the compounds singly did not have a detrimental effect on hepatocyte biology, but in combination they displayed a more marked effect. Following exposure, female hepatocytes displayed greater cell number, induced caspase 3/7 activity and lower ATP levels than male hepatocytes. This suggested that female hepatocytes were likely undergoing apoptosis during cell dedifferentiation. Whereas, male hepatocytes appeared to be necrotic as they detached from the extracellular matrix. Whether these observations are a consequence of different levels of metabolic enzyme function in the hepatocyte populations, or the effects manifest due to other sex-dependent processes, will be the study of future experimentation.

The sex differences reported in these studies are consistent with previous studies on maternal smoking on foetal development. Recently, Filis et al. demonstrated that in male foetuses maternal smoking affected pathways regulating liver fibrosis and cirrhosis, whereas in female foetuses glucose metabolism was more affected (Filis et al. 2015). Sex-specific responses to maternal smoking is also reflected in the balance of foetal endocrine signals (O’Shaughnessy et al. 2007, 2011) and in the development of other organs, including the gonads (O’Shaughnessy et al. 2007; Fowler et al. 2014; Drake et al. 2015) and the placenta (Gabory et al. 2013). While sexual dimorphism exists in the expression of these pathways, there are also studies which indicate sex-independent responses leading to disease in the adult (Allina et al. 2011).

In summary, our approach has shown that pluripotent stem cell derived hepatoblasts and hepatocytes represent a useful tool to model foetal liver biology ‘in the dish’, providing valuable information on sex differences that occur following exposure to components of cigarette smoke.

## Electronic supplementary material

Below is the link to the electronic supplementary material.

**Supplementary Table 1**. Antibodies employed in immunofluorescence studies (DOCX 12 kb)

**Supplementary Fig. 1.** Dose response curves to smoking derivatives in female hepatocytes upon exposure. Female hepatocytes were incubated for 8 days with different concentrations of the smoking derivatives; upon exposure, levels of expression of the cell health markers ATP and Caspase 3/7 and the cell function CYP1A2 and CYP3A were measured. The dotted lines indicate the calculated IC_50_ value for all the measurements (TIFF 33094 kb)

**Supplementary Fig. 2.** Dose response curves to smoking derivatives in male hepatocytes upon exposure. Male hepatocytes were incubated for 8 days with different concentrations of the smoking derivatives; upon exposure, levels of expression of the cell health markers ATP and Caspase 3/7 and the cell function CYP1A2 and CYP3A were measured. The dotted lines indicate the calculated IC_50_ value for all the measurements (TIFF 33458 kb)
