# Supplementary material for: Modelling foetal exposure to maternal smoking using hepatoblasts from pluripotent stem cells
Source: Arch Toxicol. 2017 May 16;91(11):3633–43. doi: 10.1007/s00204-017-1983-0 (PMC5696490; doi:10.1007/s00204-017-1983-0)
Supplement: Supplementary file 1 — Supplementary Table 1. Antibodies employed in immunofluorescence studies (DOCX 12 kb) [file 204_2017_1983_MOESM1_ESM.docx]

**Supplementary Table 1.**

| **Antigen** | **Manufacturer** | **Catalogue**  **Number** | **Specie** | **Dilution** |
| --- | --- | --- | --- | --- |
| HNF4α | Santa Cruz | SC-8987 | Rabbit | 1:100 |
| Albumin | Abcam | AB10241 | Mouse | 1:100 |
| E-Cadherin | Abcam | AB1416 | Mouse | 1:100 |
| Zonula Occludens-1 (ZO-1) | Abcam | AB59720 | Rabbit | 1:200 |
| Alexa Fluor 568 anti rabbit | Life Technologies | A10042 | Donkey | 1:400 |
| Alexa Fluor 488 anti mouse | Life Technologies | A11001 | Goat | 1:400 |
| Alexa Fluor 488 anti rabbit | Life Technologies | A150077 | Goat | 1:400 |
