# Supplementary figures and images for: Modelling foetal exposure to maternal smoking using hepatoblasts from pluripotent stem cells
Source: Arch Toxicol. 2017 May 16;91(11):3633–43. doi: 10.1007/s00204-017-1983-0 (PMC5696490; doi:10.1007/s00204-017-1983-0)

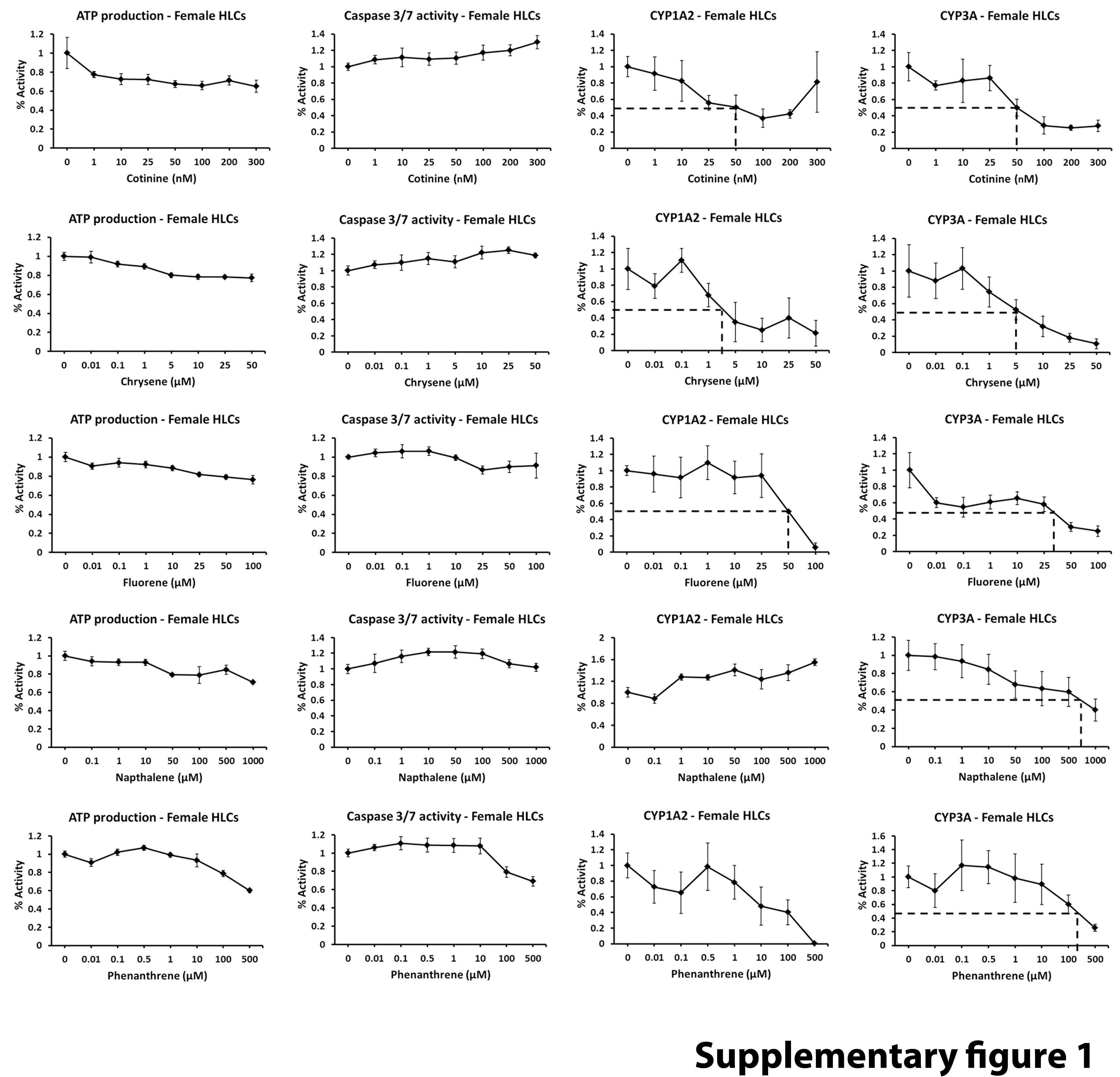

Supplement: Supplementary file 2 — Supplementary Fig. 1. Dose response curves to smoking derivatives in female hepatocytes upon exposure. Female hepatocytes were incubated for 8 days with different concentrations of the smoking derivatives; upon exposure, levels of expression of the cell health markers ATP and Caspase 3/7 and the cell function CYP1A2 and CYP3A were measured. The dotted lines indicate the calculated IC50 value for all the measurements (TIFF 33094 kb) [file 204_2017_1983_MOESM2_ESM.tif]

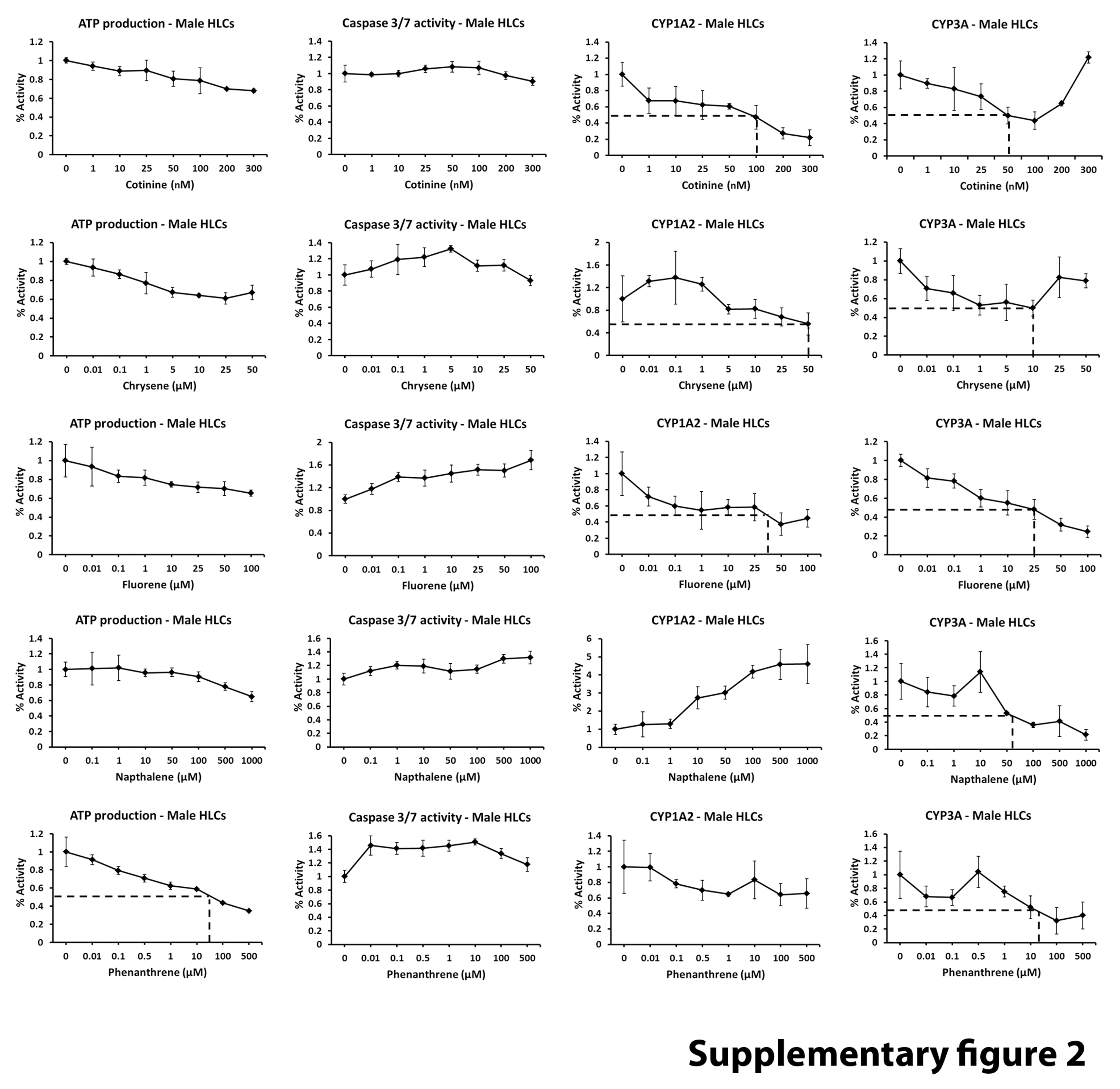

Supplement: Supplementary file 3 — Supplementary Fig. 2. Dose response curves to smoking derivatives in male hepatocytes upon exposure. Male hepatocytes were incubated for 8 days with different concentrations of the smoking derivatives; upon exposure, levels of expression of the cell health markers ATP and Caspase 3/7 and the cell function CYP1A2 and CYP3A were measured. The dotted lines indicate the calculated IC50 value for all the measurements (TIFF 33458 kb) [file 204_2017_1983_MOESM3_ESM.tif]
